# Entrainment and Modulation of Gesture–Speech Synchrony Under Delayed Auditory Feedback

**DOI:** 10.1111/cogs.12721

**Published:** 2019-03-18

**Authors:** Wim Pouw, James A. Dixon

**Affiliations:** ^1^ Center for the Ecological Study of Perception and Action University of Connecticut; ^2^ Department of Psychology, Education& Child Studies Erasmus University Rotterdam

**Keywords:** Hand‐gesture, Speech, Synchrony, Delayed auditory feedback, Cross‐wavelet analysis

## Abstract

Gesture–speech synchrony re‐stabilizes when hand movement or speech is disrupted by a delayed feedback manipulation, suggesting strong bidirectional coupling between gesture and speech. Yet it has also been argued from case studies in perceptual–motor pathology that hand gestures are a special kind of action that does not require closed‐loop re‐afferent feedback to maintain synchrony with speech. In the current pre‐registered within‐subject study, we used motion tracking to conceptually replicate McNeill's (1992) classic study on gesture–speech synchrony under normal and 150 ms delayed auditory feedback of speech conditions (NO DAF vs. DAF). Consistent with, and extending McNeill's original results, we obtain evidence that (a) gesture‐speech synchrony is *more* stable under DAF versus NO DAF (i.e., increased coupling effect), (b) that gesture and speech variably entrain to the external auditory delay as indicated by a consistent shift in gesture‐speech synchrony offsets (i.e., entrainment effect), and (c) that the coupling effect and the entrainment effect are co‐dependent. We suggest, therefore, that gesture–speech synchrony provides a way for the cognitive system to stabilize rhythmic activity under interfering conditions.

## 1 Introduction

Speech and hand gesture are seamlessly coordinated. This coordination is found on the semantic level, where gesture can mirror or augment with iconic reference what is said in speech. This coordination is also observed on a prosody level, such that the intensity changes of gesture and speech covary. Indeed, research combining motion‐tracking methodology and speech analysis is beginning to confirm that the energetic patterns of gesture and speech are flexibly and tightly synchronized (Chu & Hagoort, 2014; Danner, 2017; Danner, Barbosa, & Goldstein, 2018; Krivokapić, Tiede, & Tyrone, 2017; Krivokapić, Tiede, Tyrone, & Goldenberg, 2016; Leonard & Cummins, 2010; Parrell, Goldstein, Lee, & Byrd, 2014; Rochet‐Capellan, Laboissiere, Galvan, & Schwartz, 2008; Rusiewicz, Shaiman, Iverson, & Szuminsky, 2014; Treffner & Peter, 2002; Zelic, Kim, & Davis, 2015). The general finding from these studies is that energetic contrasts in gesture (e.g., gesture end‐point; peak velocity) are structurally related to prosodic contrasts (e.g., peak pitch; stressed syllable), which are the energetic contrasts in speech. Although evidence for entrained gesture–speech rhythms is largely based on repetitive pointing‐, beat‐tapping, or finger‐tapping gestures, where speech and gesture are scripted and produced on command (but see Danner et al., 2018), the findings appear generalizable to more spontaneous and semantically rich gestures on the basis of careful (but subjective) analysis of video recordings (e.g., McClave, 1994; McNeill, 1992, 2005; Loehr, 2004, 2012; for an overview see Wagner, Malisz, & Kopp, 2014). Given this growing evidence for the entrainment of gesture–speech rhythms, the question arises of *how* and *why* gestures are so closely controlled with respect to the rhythm of speech (Esteve‐Gibert & Guellaï, 2018; Iverson & Thelen, 1999; Rusiewicz, 2011; Rusiewicz & Esteve‐Gibert, 2018; Wagner et al., 2014).

A remarkable case study that has left a lasting theoretical imprint on how gesture researchers think about the perceptual‐motor control of hand gestures is the gesturing ability of Ian Waterman (IW) (Gallagher, 2005; McNeill, 2005; McNeill, Quaeghebeur, & Duncan, 2010; ). Since early adulthood, IW has suffered from the absence of proprioception from the neck down, which makes instrumental actions (e.g., picking up objects) practically impossible without continuous visual feedback. Without visual control, IW simply does not know where his limbs are located, let alone whether a grasp is successfully unfolding. Yet, IW produces typical looking gestures when his view of his body is blocked, sometimes without any intention or awareness of doing so. Researchers studying IW have concluded that his non‐visually guided gestures are impaired when topokinetic accuracy is required (e.g., tracing out an imagined triangle in the air), but they are otherwise largely unaffected (but see McNeill et al., 2010 and Gallagher, 2005 for a more detailed description). Most important, these researchers also concluded that IW's gestures are produced in synchrony with his speech (see Dawson & Cole, 2010 for a video example of IW's gesticulation). This finding has led these researchers to conclude that gestures must be controlled in a different way than instrumental actions (Gallagher, 2005; McNeill, 2005), which invokes the idea that gesture does not require the so‐called closed‐loop control; it does not require continuous causal influences from perception (where are my hands now/what effects do my actions have) and action (where do my hands go) to maintain gesture–speech synchrony. The idea that gestures are somehow different from instrumental actions has further been argued for on the basis of research showing that pantomimes are sensitive to visual illusions but instrumental actions are not (e.g., Westwood, Heath, & Roy, 2000), as well as by case studies of subjects with congenital phantom limbs who report gesturing with otherwise passive phantom limbs (e.g., phantom arms do not swing during walking; Ramachandran, Blakeslee, & Shah, 1998).

Yet it has also been shown that gesture–speech synchrony remains relatively stable under perturbations of hand movements or speech production, suggesting continuous *bidirectional* coupling of gesture and speech (Chu & Hagoort, 2014; McNeill, 1992; Rusiewicz et al., 2014). Chu and Hagoort (2014) found that when visual feedback of a pointing gesture is disrupted, speech will halt so as to synchronize with the perturbed (and therefore delayed) pointing movement. More specifically, Chu and Hagoort (2014) found that in a virtual environment, when the visual feedback of the pointing gesture was delayed with 117 or 417 ms (Experiment 1), or when visual feedback was suddenly horizontally displaced, removed, or put to a halt while the real gesture was ongoing (Experiments 3 and 4), gesture execution time was delayed and so was speech onset time. Gesture–speech synchronization was thus maintained. Importantly, perturbing the gestures affected speech even in the very late phases right before the onset of speech (as short as an estimated 99 ms), suggesting that interaction between speech and gesture does not become impossible while the gesture is in its execution; that is, gesture and speech do not become “ballistic” at some point (Levelt, Richardson, & La Heij, 1985).

In the final fifth experiment, instead of a *gesture* perturbation, *speech* was perturbed by changing the color of the to‐be‐referenced light when a gesture was already initiated. Participants then needed to change their speech intention, for example, from saying “this blue light” to saying “this yellow light.” As in the previous experiments, the perturbation was administered at early and late phases in the gesture execution. This way the speech intention needed be readjusted while a gesture was already underway. Such speech perturbation indeed leads to speech onset delays. Importantly, delaying speech in this way also delayed gesture execution times in early and late phases. This study shows that, at least for these pointing gestures, continuous bidirectional feedback is utilized to maintain gesture–speech synchrony. In a recent extension of this virtual reality (VR) paradigm, it was shown that visual feedback of gestures is utilized for maintaining gesture–speech synchrony even when gestures reserve degrees of freedom for iconic expression (M. Chu & P. Hagoort, unpublished data). For example, delayed visual feedback led to speech readjustments when pointing gestures traced the outline of a visually presented object. Speech was also affected by visual feedback disruptions when gestures mimed grabbing an object that was presented in VR. This suggests that even when externally controlled gestures are iconic (i.e., pantomime, iconic tracing), visual feedback affects gesture execution. In sum, the meticulous experiments by Chu and Hagoort weaken the case that gesture–speech synchrony is not regulated by perceptual feedback of actions in typical populations (in contrast to IW's case).

McNeill's (1992) classic studies assessed whether participants’ gesture–speech synchrony was affected when speech was disrupted by a delayed auditory feedback manipulation (DAF). When auditory feedback from speech is delayed by about 75–200 ms, speech becomes noticeably disfluent and slurred, and more frequent speech errors (e.g., repetition of phonemes) and slower speech rates are observed (Sasisekaran, 2012; Stuart, Kalinowski, Rastatter, & Lynch, 2002; see demonstration of the DAF effect from our previous exploratory study https://osf.io/5h3bx/). However, despite obtaining a classic DAF effect when participants retold a cartoon they had just watched, McNeill (1992) reported that gesture–speech synchrony was completely unaffected. In a second experiment, however, McNeill noted that gesture–speech synchrony was noticeably affected when participants had to recite memorized sentences and gesture movements. This suggests that there might be an important role for spontaneity in gesture–speech synchrony (cf., Chu & Hagoort, 2014). McNeill's (1992) findings on the robustness of gesture–speech synchrony is further supported by comparable classic research, showing that gestures are suspended, halted or lengthened when involuntary speech disfluencies occur (e.g., fillers like “uh”; stuttering; word repetitions; e.g., De Ruiter, 1998; Seyfeddinipur, 2006).

Detailed investigation of the kinematics of gesticulation under DAF obtained mixed results, however. Rusiewicz et al. (2014) used a task in which participants pointed and verbally labeled targets. In the DAF condition (half of the trials), participants heard their own speech with a 200 ms delay, which resulted in elongated spoken responses. In contrast to McNeill's (1992) original DAF study, *increased* gesture–speech *asynchrony* was found for DAF vs. NO DAF by Rusiewicz et al. (2014). That is, the time difference of the gesture–launch midpoint and the vowel‐to‐vowel midpoint of the referenced target word increased under DAF. Although there were more attempts at resynchronization as gestures were lengthened under the DAF condition, these results were not statistically reliable. However, in a recent experiment by Chu and Hagoort (unpublished data; Experiment 3), inducing a 100 ms DAF did lead to reliable slowing down of pointing and tracing gestures, although differences in gesture–speech synchrony were not assessed as such. To sum up, these detailed kinematic studies on externally directed pointing gestures suggest that such gestures are indeed reactive to DAF, but also leave unclear whether gestures *completely* resynchronize with perturbed elongated speech under DAF (cf., Kelso, Tuller, Vatikiotis‐Bateson, & Fowler, 1984).

### 1.1 Open research questions

There are still several key open questions about the stability of gesture–speech synchrony under conditions of perturbation and the precise role that perceptual feedback plays in maintaining stability. First, perturbation research with relatively high kinematic detail has solely focused on gesture–speech synchrony of (pointing) gestures that are directed toward a visually available object (Chu & Hagoort, 2014, unpublished data; Rusiewicz et al., 2014). These types of gestures are an exotic member of the family of gesture as they are exogenously controlled with respect to an external target. Such externally directed gestures are thus closely related to instrumental actions in this respect. Therefore, it is not certain that more fluid and spontaneous gestures (e.g., beat and iconic gestures) produced during narration will show similar dynamics (cf., Ian Waterman). Importantly, McNeill's (1992) original descriptive report does suggest that gesture–speech synchrony is maintained under DAF, even when it concerns spontaneous gestures produced during narration. However, as promising as these results are, more empirical detail is required to fully understand how gesture–speech synchrony is affected by DAF for spontaneous gesturing (as also argued by Rusiewicz et al., 2014). As such, we aim to conceptually replicate McNeill's (1992) classic setup by assessing the effect of DAF during narration of a cartoon while objectively tracking movement and speech with high resolution.

In addition to the issue of *whether* gesture is reactive to speech perturbation, we aim to address the question how and why it is reactive. It has been argued from a dynamical systems perspective of gesture (McNeill, 1992, 2005; Rusiewicz et al., 2014; see also Rusiewicz et al., 2011) that the stability of gesture–speech synchrony under DAF suggests that gesture and speech flexibly organize into equivalent functional sensorimotor solutions through tight bidirectional coupling of the systems. This stability is likened to the classic finding by Kelso et al. (1984), who showed that when the jaw is locked in the midst of articulating a syllable, the lower lip *or* tongue spontaneously jumps into place to successfully complete the syllable (depending whether the perturbation is successfully resolved by a lip or tongue intervention). Yet, in the case of *gesture–speech synchrony* under DAF, it could still be that stability is maintained *because* the delayed auditory feedback only affects speech and *not* gesture. That is, *if* the gesture system is merely uni‐directionally governed by speech production and is not coupled to perceptual auditory feedback, it will always be produced in synchrony with speech. Stability, under such a view, is maintained because the gesture system is ignorant with respect to the auditory shadow of speech; the gesture system is informationally closed in this respect (a feature that is central to information‐processing theories of gesture; De Ruiter, 2000).

We think that a dynamically coupled gesture–speech system (as opposed to an informationally closed system) would be reactive to DAF in a different way than initially conceived by proponents of a dynamical systems approach to gesture (McNeill, 1992; Rusiewicz et al., 2014). Namely, in line with the concept of entrainment as described by Rusiewicz et al. (2014; see also Iverson & Thelen, 1999; Rusiewicz, 2011), coupled oscillators will diverge from their individually preferred rate of oscillation and will form a new stable oscillation pattern. This joint preferred oscillation pattern will function as a stable point attractor, such that the system will return to this stable state even when one of the subsystems is perturbed (i.e., stability of gesture–speech synchrony). Under this view, given ordinary gesture–speech synchrony, *two* oscillators, one for speech and the other for gesture, are dynamically or “weakly” coupled. However, under DAF, there is a third oscillator introduced in the form of a delayed feedback. If the DAF signal functions as a third oscillator, it should serve as a rhythmic attractor for both speech and gesture. Given that speech and gesture have their own preferred rate of oscillation, it is also likely that DAF will affect speech and gesture differently. Note that this possibility is not far‐fetched given that it is well known that humans naturally and involuntarily tune their actions (e.g., finger tapping) to auditory rhythms (e.g., metronome; for an overview, see Port, 2003; Repp, 2005). In sum, stability under perturbation is a necessary but not sufficient condition for arguing for the dynamic coupling of gesture and speech, as accounts that posit that gesture couples with speech production (and not speech feedback) would equally predict gesture–speech synchrony under DAF. Rather, entrainment of speech *and gesture* to the DAF signal is also to be predicted if the gesture–speech system is a dynamical system. This study examines how coupling and entrainment might account for DAF effects in gesture–speech synchrony. Further, we explore the implications of the dynamical systems account for understanding the function of gesture‐speech synchrony for the communication system.

### 1.2 Summary model predictions

**Figure 1:**
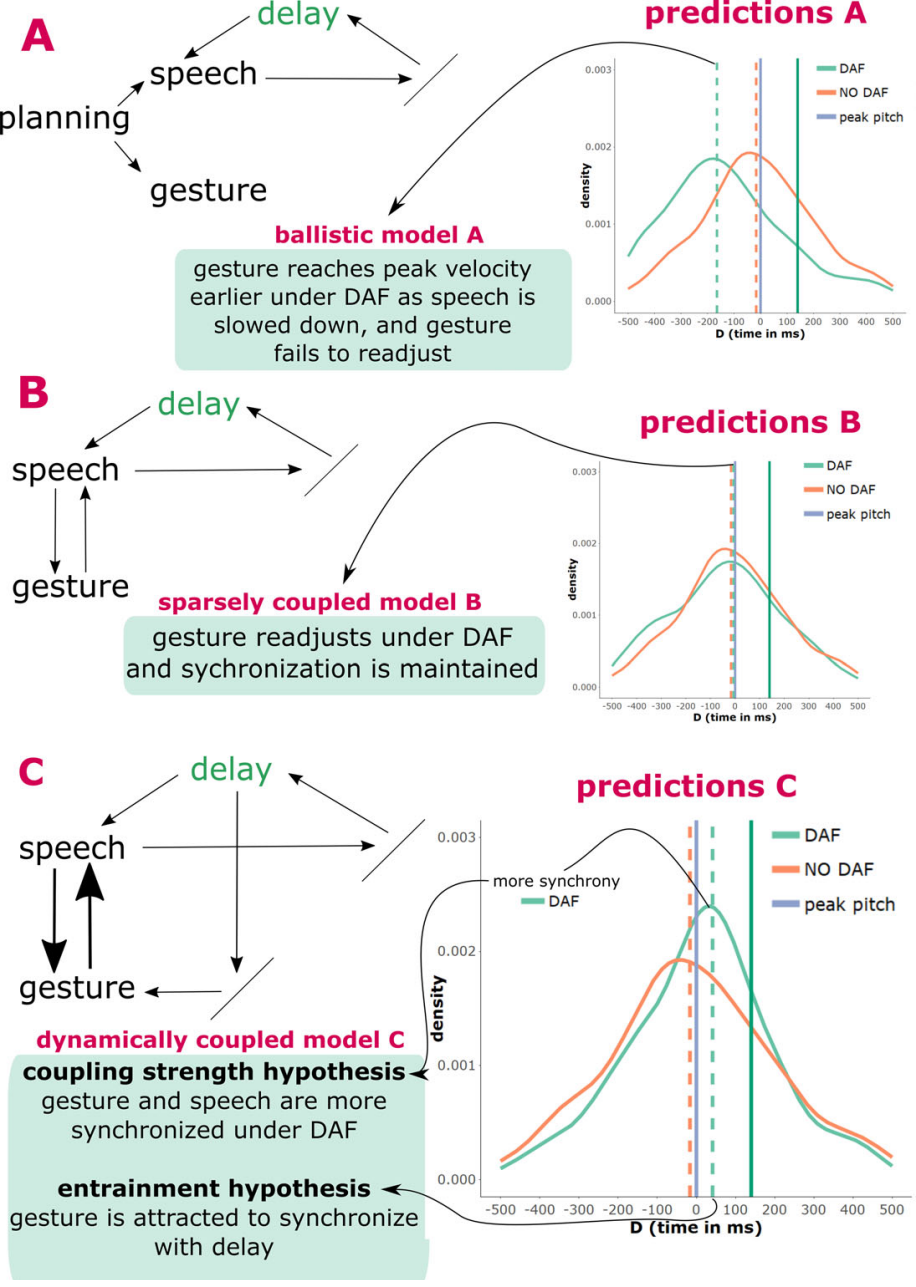
Model predictions. *Notes*. On the left‐hand side a schematic of the ballistic model (a), the sparsely coupled model (b), and the dynamically coupled model (c) are provided. On the right‐hand side, predictions for each model are given by frequency distributions of gesture‐speech synchrony expressed by *D* (temporal *D*ifference between peak velocity gesture and peak F0 speech). The blue vertical line at *D* = 0, is the reference point and indicates the moment where the peak F0 is reached. The solid green vertical line at *D* = 140 indicates when the DAF loop returns a peak F0 to the speaker (i.e., the length of the auditory delay). The striped vertical lines indicate the average synchrony for gestures produced under NO DAF (orange) and DAF (light green). When distributions are shifted to the left, this indicates that gesture's peak velocity occurred before peak in F0 (i.e., gesture reaches peak earlier than speech); this is predicted for gestures under DAF by a ballistic model. A more peaked frequency distribution indicates that gesture–speech is less variably aligned with speech (more coupled); this is predicted by the coupling strength hypothesis of the dynamically coupled model. When distributions are shifted to the right, this indicates that gestures are completing their peak velocity later in time with respect to peak F0; this is predicted for gestures under DAF by the entrainment hypothesis.

Our previous review of the different predictions that arise out of the literature can be summarized by three models that each have their separate predictions of how gestures behave with respect to speech under a DAF manipulation (see Fig. 1).

On the one hand, we have the *ballistic model* (model A in Fig. 1), which suggests that gesture and speech are decoupled in the execution phase and therefore any perturbation in speech production (due to DAF) will not affect gesture. This model predicts that a gesture will therefore be executed as planned and will reach its kinematic peaks (e.g., peak velocity) earlier than usual, because speech is slowed down due to DAF. Thus, the ballistic model predicts that gestures will come to lead speech in time (i.e., speech will lag behind gesture).

What we call the *sparsely coupled model* (McNeill, 1992; Rusiewicz, 2011) assumes that gesture can readjust its trajectory because there is continuous bidirectional feedback between gesture and speech (even at late phases of execution). This sparsely coupled model thus predicts that gesture–speech synchrony is maintained under DAF vs. NO DAF.

Our newly proposed *dynamically coupled model* (C) has two alternative predictions. First, it predicts that gesture–speech synchrony can be modulated under DAF so as to resist perturbation. As such, we predict that gesture–speech synchrony is more pronounced under DAF versus NO DAF (referred to as the “coupling strength hypothesis [CSH]”). Second, given that we assume that the gesture system is also reactive to the perceptual delayed feedback of speech, it is predicted that the gesture system will be slightly attracted to synchronize with delayed speech. This “entrainment hypothesis [EH]” entails that gesture‐speech synchrony is recalibrated and gesture accommodates to the interfering DAF oscillator, meaning that gesture is more likely to follow speech as to align more with delayed speech.

### 1.3 Current paradigm

In our previous exploratory DAF study with four participants who produced 275 gesture events (for full analyses report, see Pouw & Dixon, unpublished data), we replicated the original experiment by McNeill (1992) with a few modifications. After watching a cartoon, four participants retold the plot, under alternating bouts of DAF and NO DAF. In contrast to McNeill (1992), we tracked the motion of the dominant hand (at 240 Hz) to record gestural movements, which allowed us to identify energetic peaks during each gesture event (peak velocity, peak acceleration, peak deceleration). Gesture events and gesture types (beat and iconic) were identified using ELAN and the motion‐tracking time series (Crasborn, Sloetjes, Auer, & Wittenburg, 2006; Lausberg & Sloetjes, 2009). We extracted the Fundamental Frequency (F0; perceived as “pitch”) from the audio (using PRAAT, Boersma, 2001), so as to identify peaks in pitch within relevant gesture–speech events.

In this exploratory study, we obtained preliminary results that aligned with the dynamically coupled model. More precisely, for beat gestures, peak velocity (as a kinematic anchor point for gesture) had lower absolute deviances from peak pitch under DAF vs. NO DAF, suggesting stronger synchrony of gesture and speech under DAF (CSH). Secondly, we found for beat gestures a promising indication that the key kinematic events (gesture onset, peak acceleration, peak velocity, peak deceleration) of gestures were consistently positively shifted under DAF as compared to NO DAF with about 34 ms (EH), suggesting that gestures are attracted toward synchronizing with the auditory shadow of speech.

In this study, we replicated the research setup on the basis of our results from the exploratory study. We pre‐registered analysis plans (see https://osf.io/pcde3/) for two confirmatory analyses (based on the promising results in the exploratory study), in which we planned to assess whether beat gestures are as follows: (a) more stably coupled under DAF vs. NO DAF, as indicated by lower mean deviances of peak velocity from peak pitch (CSH), and (b) whether there is a consistent positive shift for kinematic anchor points (gesture onset, peak acceleration, peak velocity, and peak deceleration) relative to peak pitch suggesting that gesture entrains to the DAF signal (EH). We also performed several exploratory analyses assessing whether these effects hold for iconic gestures, and what the relationship is between coupling strength and degree of entrainment.

We further explored the CSH using cross‐wavelet analysis (see e.g., Romero et al., 2018) wherein we assessed how the temporal structure of speech and gesture is correlated under DAF versus NO DAF on *multiple nested time‐scales*. Several theorists have maintained that gesture and speech operate on nested timescales, suggesting that it is an oversimplification to think of gesture–speech events as isolatable units of communicative expression (Kendon, 2004; McNeill, 2005). For example, multiple sequenced beat gestures may move with the rhythm of speech (operating on the scale of milliseconds; Leonard & Cummins, 2010); gesture sequences may reflect syntactic conventions that are present on the verbal sentential level (seconds; Kita & Ozyurek, 2003). Furthermore, some gestures seem to retain similarity in their kinematic profiles as they recur at several times in a discourse, providing anchors for discourse cohesion (minutes; McNeill et al., 2002). As such, if gesture and speech differ in their coupling under DAF vs. NO DAF, such changes in correlation between temporal coupling of gesture and speech might be present on the multiple time scales (e.g., phonemes to syllables to sentences). To quantitatively assess the shared temporal structure of speech and gesture, we related manual movement (velocity) with the *amplitude envelope* of speech (henceforth ENV). ENV is a continuous measure for tracking the rhythmicity of speech and correlates highly with articulatory movements (Chandrasekaran, Trubanova, Stillittano, Caplier, & Ghazanfar, 2009).

## 2 Method

### 2.1 Sample and design

The current sample consisted of ten undergraduate students from the University of Connecticut (7 males; 8 right‐handed, 2‐left handed; Age *M* [*SD*]* *= 18.8 [0.79] years). Two participants of the original sample were excluded because they did not gesture. We therefore tested an additional two participants so as to reach the sampling plan of 10 participants.^1^ All but one participant were native speakers of American English. One participant reported Spanish as her native language but had 19 years of experience with speaking American English. This study entails a within‐subjects design with one factor with two levels: DAF versus NO DAF. The current sample generated 25.73 min of narration (11.00 min DAF vs. 14.73 min NO DAF) containing over 500 gesture events.

### 2.2 Apparatus

#### 2.2.1 Motion tracking

To track hand movements, we used a Polhemus Liberty (Polhemus Corporation, Colchester, VT, USA) to collect 3D position data at 240Hz (~0.13 mm spatial resolution) with a single motion‐sensor attached to the top of the index finger of the dominant hand. Thus, the position data are determined by movements of the arm, wrist, and finger. We only allowed for and recorded the motion of the dominant hand (rather than both hands) to simplify interpretations regarding gesture–speech synchrony.

#### 2.2.2 Audio

We obtained speech data by using an RT20 Audio Technica Cardioid microphone (44.1 kHz).

#### 2.2.3 Camera

Participants were video recorded (29.97 fps) using a Sony Digital HD Camera HDR‐XR5504 recorder.

#### 2.2.4 Motion and audio recording

We used a C++ script made publicly available by Michael Richardson (Richardson, 2009) to collect movement data, which we further modified to simultaneously call and write audio data using scripts to enable recording of sound from a microphone (using toolbox SFML for C++ https://www.sfml-dev.org/).

#### 2.2.5 Delayed auditory feedback

**Figure 2:**
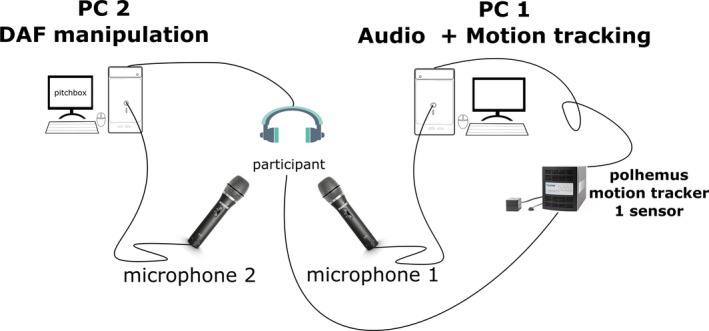
Schematic overview setup. *Note*. Two PCs were used, one that handled the recording of audio and motion tracking of the dominant hand (PC 1) and PC 2 recorded audio and played back the audio to the participant at a 150 ms delay (DAF manipulation).

For the delayed auditory feedback manipulation, we used a second microphone and wireless headphone connected to a separate PC (see a Fig. 2 for how the different systems are used in this study). We used open software called *Pitchbox 2.0.2* (Juillerat, n.d.) to delay feedback with a microphone. This software was originally developed to modify acoustics, such as shifting the pitch of voice, but in the “normal” mode it will produce a latency between voice production and acoustic output. This degree of latency is dependent on the computer system's hardware specifications. We pre‐tested the latency between microphone input and audio output that was produced when running it on a laptop, and we obtained that the latency was consistently between 130 and 150 ms for this system. This range for testing the auditory feedback delay phenomenon lies well above the lowest DAF manipulations that have been known to induce a DAF effect (e.g., Stuart et al., 2002). The effect of DAF was indeed very noticeable (see sample of speech and gesture under DAF for the exploratory study, using identical equipment as the current confirmatory study: https://osf.io/5h3bx/).

### 2.3 Procedure

Participants were equipped with a glove for the dominant hand, which allowed attachment of the Polhemus motion sensor with Velcro tape. In previous exploratory studies, we observed that this glove did not restrict spontaneous gesturing rates (Pouw & Dixon, 2018). The glove was attached before watching the video so that the subject got used to wearing it. In the current experiments, participants first watched the cartoon “Canary Row” lasting about 350 s (e.g., McNeill, 1992, 2005). Participants were informed they would retell the cartoon narrative to the experimenter later on. After watching the cartoon, participants were familiarized with the DAF manipulation. The experimenter further explained that during narration the DAF would sometimes be turned on, but that participants would need to keep narrating as best as they could. In the narration phase, for the first 30 s participants always narrated without DAF manipulation (warm‐up phase). After the warm‐up phase, the DAF was turned on or turned off (depending on counterbalanced condition) every 60 s. We counterbalanced DAF timing as it is possible that otherwise DAF would occur for a particular segment of the cartoon narrative and thus perhaps for particular types of gesture–speech events. No instructions were given about whether to use hand gestures.

### 2.4 Data preparation

The first author transcribed speech and identified gesture events using the annotation software ELAN (Lausberg & Sloetjes, 2009). We also loaded the motion‐tracking time series into ELAN to manually determine the onset and end‐phase of the gestures (see Crasborn et al., 2006); the video data were used to determine the type of gestures (beat vs. iconic^2^ vs. undefined gestures). The gesture was marked as ending at the point at which the gesture completed its main stroke. Thus, we did not include a pre‐stroke hold, post‐stroke hold, nor a retraction phase, if present (see Kita, van Gijn, & van der Hulst, 1998).

Similar to the procedure used by Pouw and Dixon (2018), peak velocity, peak acceleration, and peak deceleration were determined with respect to peak pitch (see code online). We applied a low‐pass first‐order Butterworth filter to the position velocity traces with a cut‐off of 33 Hz.^3^

**Figure 3:**
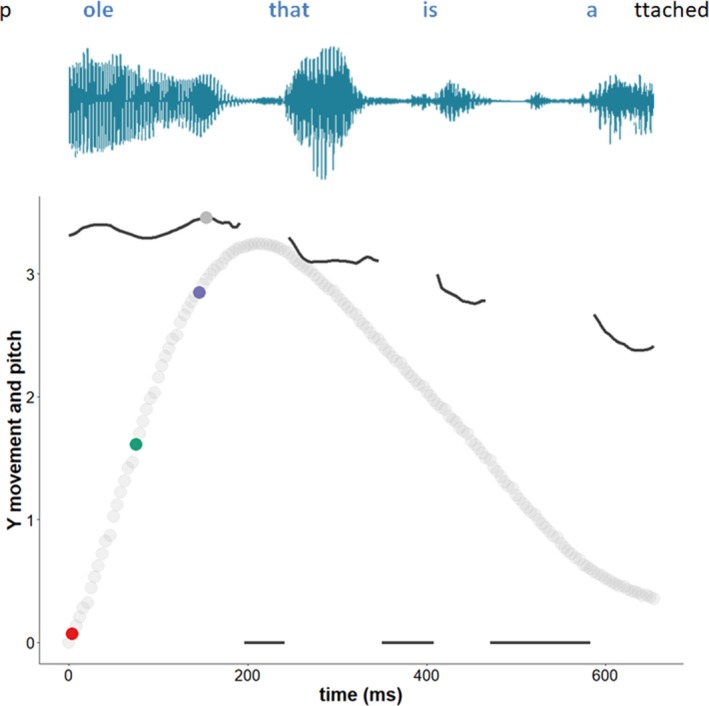
Example gesture and F0 time series and energetic peaks. *Notes*. Vertical motion of the hand (Y movement) and F0 (pitch track) time series segment. We have superimposed the raw sound waveform in blue above. The F0 values are rescaled for this example and reflect the opening of the vocal folds for the phonated parts of the speech segments. The colored dots reflect the key energetic peaks; solid gray dot = peak pitch, red dot = peak acceleration, green dot = peak velocity, and purple dot = peak deceleration. This data example is based on an iconic gesture produced under NO DAF in the exploratory study (see video clip at https://osf.io/ax48y/). The participant traces out the outline of a pole while saying “pole that is attached to the building,” wherein the gesture overlaps with the blue segment of speech. In this example, it is clear that energetic peaks are situated in the beginning phase of the gesture, which coincides with the peak in pitch (and the semantically relevant part “pole”).

With regard to taking peak F0 as a speech anchor point, it should be noted that there are many other viable anchor points for speech which could be used in gesture–speech synchrony analyses. Rochet‐Capellan et al. (2008) used the maximum extension of the jaw during syllable pronounciation. Rusiewicz et al. (2014) used vowel–vowel midpoint of an uttered target word. Additionally, one could use regions of prosodic contrast as indexed by Tones and Breaks Indices (ToBi) prosody analyses (e.g., Danner, 2017; Loehr, 2004; Shattuck‐Huffnagel, 2018). We chose however to use peaks in F0 near a gesture kinematic peak, as all types of pitch accents (e.g., ToBi typology) are at least characterized by sudden peaks in F0. Furthermore, Krivokapić et al. (2016) have compared different prosodic properties as they relate with gesture, and they found preliminary evidence that gesture is more likely coordinated with F0 modulations (rather than, e.g., articulatory lengthening). Our current method of taking peak F0 as an anchor, further provides an objective automatable method suitable for non‐scripted speech that replaces otherwise laborious inter‐rater judgments (ToBi) and context specific measures (e.g., vowel‐to‐vowel midpoint is only relevant if there are two vowels) that are often employed in scripted and controlled contexts (Rusiewicz et al., 2014) or otherwise applied for small amount of data (e.g., Loehr, 2004; Shattuck‐Huffnagel, 2018). Finally, note that we will also corroborate our F0 analyses by using another speech property that we will relate to gesture, namely the amplitude envelope of speech (see below; Fig. 3).

#### 2.4.1 Inter‐rater reliability gesture annotation

To assess the reliability of distinguishing between beat and iconic gesture events, a second rater annotated beat and iconic gestures for 20% of the data (for the first two participants). We computed reliability scores based on time windows of the second coder which overlapped with at least 50% of that with the first rater. The raw global agreement was initially 80.06% and a modified kappa of 0.57 (“moderate agreement” according to Landis & Koch, 1977). However, since this was lower than the ideal modified Kappa coefficient of .75 (“substantial agreement”; Landis & Koch, 1977) that we aimed for, the first author and the second rater discussed disagreement for the first 20% of the data and the second‐rater recoded another 20% of the data (for the last two participants). This improved reliability to modified kappa of 0.69 (raw global agreement of 89.47%). Given that this 0.69 modified kappa approached 0.75 and is in the range of 0.61–0.81 for “substantial agreement” (Landis & Koch, 1977), we decided to proceed and base our analyses on first rater's coding for all the data analyses. During inspection of the gesture codings in ELAN, after the first and second round of inter‐rater comparisons, we observed two sources of discrepancies. First, discrepancy lay in determining whether a gesture is a single event or is a part of a sequence of gestures (especially for beat gestures which sometime beat in rapid sequential fashion). Second, there were some divergences on whether a gesture was of a beat or iconic nature.

#### 2.4.2 Speech F0

We extracted F0 (or pitch) time series using PRAAT with a range suitable for female (100–500 Hz) or male (75–500 Hz) voice range (Boersma, 2001). We matched the sampling rate of pitch with that of the motion tracker (240 Hz: 1 sample per 4.16 milliseconds).

### 2.5 Data aggregation

Using a custom‐made code in R, the data from ELAN, PRAAT, and motion tracking were aggregated into one dataset (for a tutorial see Pouw, Trujillo, Dixon, unpublished data). We aggregated movement data with the pitch and envelope data using custom‐written R code available on https://osf.io/pcde3/. We read in ELAN gesture and speech annotation files using a custom–made script in R, so as to mark relevant movements in the time series.

### 2.6 Speech amplitude envelope for exploratory continuous analyses

**Figure 4:**
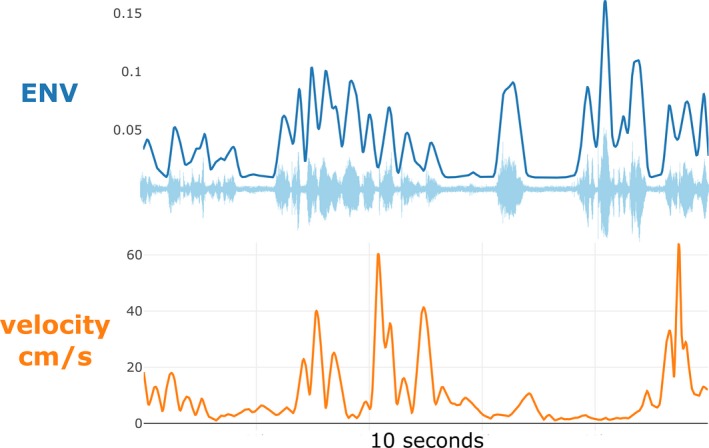
Example amplitude envelope (ENV) time series and raw audio waveform. *Notes*. Example time series (10 s narration) of the amplitude envelope (upper panel) and the raw waveform. The lower panel shows gesture velocity. Note that the ENV time series essentially traces the outline of the maximum values of the raw waveform.

A speech signal has both high‐frequency fluctuations (fine structure) and low‐frequency fluctuations. The low‐frequency fluctuations can be captured by the amplitude envelope (ENV), which can be reconstructed from the raw signal using the Hilbert transform (He & Dellwo, 2017). The amplitude envelope has been found to reliably correlate with articulatory gestures such as lip movements and is a good measure for tracking the rhythm of speech (Chandrasekaran et al., 2009; Tilsen & Arvaniti, 2013). As ENV provides a smoother and more continuous time series than pitch track (as F0 is only registering during phonation), it is more amenable for comparing the continuous rhythmic and temporal structure of gesture and speech (i.e., more suitable for cross‐wavelet analysis as introduced in the exploratory section of the Results). The current ENV time series (see Fig. 4) was produced by applying the PRAAT script by He and Dellwo (2017; see also He & Dellwo, 2016), which provides a time series in scaled Hilbert Units ranging from 0 (minimum amplitude) to 1 (maximum amplitude). This is a scaled range (per individual) and thus contains no information about between‐subject differences in amplitude.

## 3 Results

### 3.1 Descriptive: Gesture and speech rates

A total of 573 gesture events (NO DAF = 301 vs. DAF = 262) were observed (beat = 263, iconic = 260, undefined = 49), with a mean gesture frequency per minute of 21.00 (*SD *= 10.59). Table 1 provides an overview of the production rates of the different gestures, as well as speech rate (spoken words per minute narration). These findings indicate that there are no prominent differences in gesture rates depending on condition. The total amount of gestures for this dataset was considerably spread over all the participants, and all participants gestured during the task. For individual differences in gesture rates, see here: https://osf.io/n9evb/. Participants had a slower speech rate (words per minute) of about 7% in the DAF condition, confirming qualitative observations that speech was noticeably affected by the manipulation. However, the effect of DAF did seem to vary per participant, as also indicated by the high standard deviations.

**Table 1:** Mean gestures per minute, speech rates per minute speech, F0 (“pitch”) values, and narration time for each condition

Condition	Beat p/m	Iconic p/m	Undefined p/m	F0 *M* (*SD*)	Word Rate p/m Speech (*SD*)	Approx. Time of Narration
DAF	11.46	10.36	2.00	140.95 Hz (44.00)	309 (193)	11.00 min
NO DAF	9.30	9.91	1.90	145.77 Hz (49.38)	332 (102)	14.73 min

Notes

The mean gesture and speech rates (words spoken) are given *per minute* narration. Pitch (F0) values are given in Hertz. Note that narration time will be different from speech time as participants sometimes take moments to pause.

### 3.2 Descriptive: Gesture kinematics

Table 2 provides estimates for two kinematic properties per condition and gesture types. Average Jerk provides an estimate of the smoothness of the movement. Although gestures produced under DAF were generally slower, there were no pronounced differences in condition. The average time for gesture events for the NO DAF condition was 758 ms (*SD* = 431 ms, 95% CI [709, 805]) as opposed to 698 ms (*SD* = 419 ms, 95% CI [647, 749]) for the DAF condition; beat gestures (*M*_NODAF_ = 573, *SD*_NODAF_ = 256, *M*_DAF_ = 563, *SD*_*DAF*_ = 273), iconic gesture (*M*_NODAF_ = 916, *SD*_NODAF_ = 485, *M*_DAF_ = 758, *SD*_DAF_ = 375).

**Table 2:** Gesture kinematics

Condition		BEAT		ICONIC	
		Peak Velocity cm/s	Average *z* Jerk	Peak Velocity cm/s	Average *z* Jerk
DAF	*M* (*SD*)	30.33 (13.04)	−0.24(0.70)	46.90 (20.46)	0.20(0.95)
	95 CI% [lower, upper]	[28.04, 32.64]	[−0.37, −0.12]	[43.11, 50.70]	[−0.03, 0.38]
NODAF	*M* (*SD*)	32.31 (14.26)	−0.18(0.67)	48.99 (21.18)	−0.08(0.84)
	95 CI% [lower, upper]	[29.91, 34.73]	[−0.29, −0.06]	[45.53, 52.46]	[−0.05, 0.21]

Note

Average Jerk is rescaled (standardized) as value ranges are small.

### 3.3 Current Analyses: Gesture–speech synchrony

**Figure 5:**
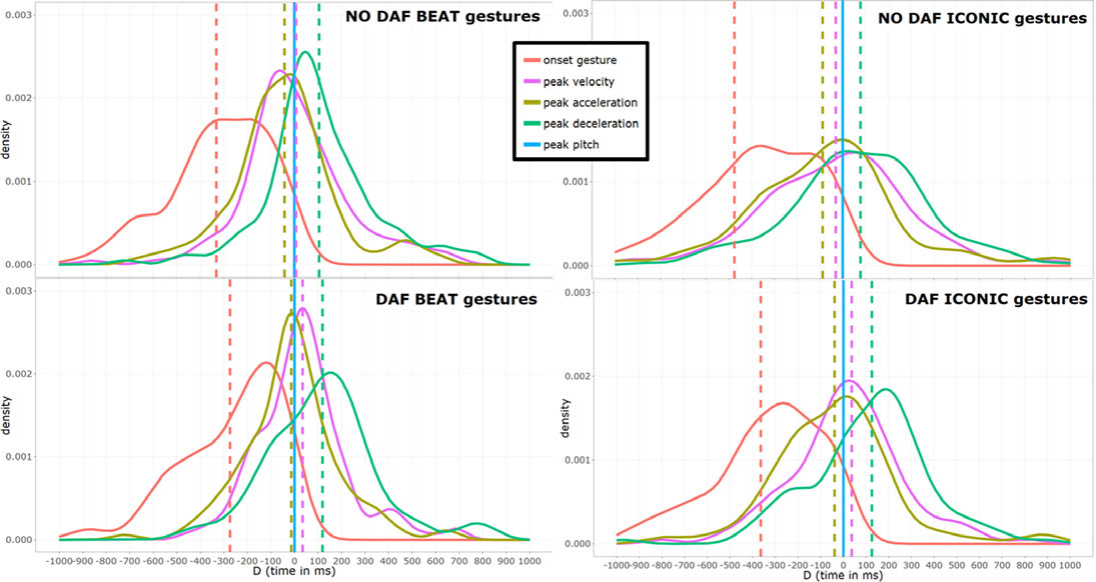
Distributions of *D* for each condition and gesture type. *Notes*. Smoothed frequency distributions of *D* for each gesture property. The difference in the timing (*D*) peak pitch relative to a gesture property is shown (blue line at zero *D*; i.e., moment of peak pitch). The peak of the distributions represents the *mode* of *D*. The dotted lines represent the *mean* of *D*. Negative‐valued *D*'s indicate that peak pitch ocurred after that gesture property. Note that for DAF gestures there seems to be a consistent positive shift of all *D* distributions, and a consistent increase in the sharpness of the peak for the distributions. Smoothed distribution plots were produced with the ggplot2 “geom_density” function. This function draws on the “1d Kernel Density Estimate” function called “stat_density” (R code available at https://osf.io/pcde3/).

The current analyses were aimed to assess gesture‐speech synchrony under DAF versus NO DAF. Table 3 and Fig. 5 show the summary statistics for these distributions. Here, the temporal differences (*D*) in milliseconds are reported for a particular kinematic property (e.g., peak velocity) occurring relative to peak pitch in speech (e.g., *D* for peak velocity = time peak pitch − time peak velocity). Consistent with the CSH, it can be seen from the graph and numeric data that, indeed, there is a lower standard deviation for *D* peak velocity for beat gestures under DAF versus NO DAF. Furthermore, consistent with the EH*,* we find that there is a positive shift for all *D*'s for gestures produced under DAF vs. NO DAF. Next, we will formally test our hypotheses with confirmatory and exploratory analyses. Note, that as stated in the pre‐registration we have restricted our alpha to .025, given that the two main confirmatory hypotheses that are tested.

**Table 3:** Mean difference *D* (peak pitch − gesture property) in milliseconds per condition

Kinematic Property	BEAT		ICONIC	
	NO DAF	DAF	NO DAF	DAF
Onset				
*M* (*SD*)	−331 (238)	−274 (227)	−475 (435)	−364 (282)
95% CI [lower, upper]	[−372, −291]	[−314, −234]	[−546, −404]	[−416, −312]
Peak acceleration				
*M* (*SD*)	−42 (223)	−14(204)	−88 (370)	−39 (275)
95% CI [lower, upper]	[−79, −4]	[−50, 22]	[−149, −28]	[−90, 12]
Peak velocity				
*M* (*SD*)	7(230)	34 (193)	−31 (379)	37 (295)
95% CI [lower, upper]	[−32, 46]	[0, 68]	[−92, 32]	[−18, 91]
Peak deceleration				
*M* (*SD*)	105 (234)	119 (234)	78 (422)	126 (291)
95% CI [lower, upper]	[66, 145]	[78,160]	[9.15, 147]	[72,180]

### 3.4 Coupling strength hypothesis

#### 3.4.1 Confirmatory analyses CSH: Beat gestures

Using a mixed regression model^4^ (nlme version 3.1‐131; random intercept for participant), we assessed differences in absolute deviances from peak pitch relative to peak velocity of beat gestures. In the exploratory study, we had found that beat gestures’ absolute deviances in peak velocity‐peak pitch timings (i.e., absolute *D* peak velocity) were lower for the DAF condition, indicating more reliable gesture‐speech coupling under DAF vs. NO DAF (Pouw & Dixon, unpublished data).

A model for absolute *D* peak velocity containing condition (NO DAF vs. DAF) did not reliably improve fit, compared to a model predicting the overall mean (change in χ^2^[1] = 1.32, *p *= .250). Model estimates did indicate that gestures produced under NO DAF had higher absolute *D* (21 ms) for peak velocity, *b *= 21.06 [95% CI: 14.75, 56.89], but this was not statistically reliable, *t*(252) = 1.15, *p *= .25. Thus, the coupling‐strength hypothesis could not be confirmed for beat gestures with the current sample.

#### 3.4.2 Exploratory analyses CSH: SD's gestures combined

An alternative and perhaps more direct way to test the coupling–strength hypothesis is assessing whether the standard deviations of *D*'s (timing of peak pitch relative to the key kinematic properties of gesture) are lower or higher under DAF versus NO DAF. Therefore, we computed standard deviations for all *D*'s (*SDD*) per condition, participant, gesture type (beat vs. iconic), and gesture kinematic property (peak acceleration, velocity, and deceleration). If gestures are more closely coupled to speech (peak pitch), then lower standard deviations (of *D*'s) are to be expected for DAF gestures as compared to NO DAF gestures; that is, less variable timing of gesture–speech peaks.

A mixed regression model (participant as random intercept) predicting *SDD* with only condition as predictor, fit better compared to a model predicting the overall mean (change in χ^2^[1] = 11.78, *p *< .001). Adding gesture type did not further improve predictability of *SDD* (change in χ^2^[1] = 1.44, *p *= .23), suggesting that both beat and iconic gestures were not different in terms of *SDD*. Adding kinematic anchor point to the previous model also did not further improve fit (change in χ^2^[1] = 3.13, *p *= .077). Model estimates for the simple model with only condition, indicated that under DAF, gestures have lower *SDD*s as compared to NO DAF with a difference in standard deviation of about Δ*SDD* = 66 ms, *b = *65.67 [95% CI: 29.47, 101.86], *t*(49) = 3.59, *p *< .001. This analyses shows that gestures’ energetic peaks under DAF are more tightly (i.e., less variably) coupled to peak pitch as compared to gestures produced under NO DAF.

### 3.5 Entrainment hypothesis

#### 3.5.1 Confirmatory analyses EH: Beat gestures

Consistent with the exploratory study, we found a positive shift in *D*'s for the DAF condition relative to the NO DAF condition (see Table 3 and Fig. 5). The model including kinematic properties fit better than a base model predicting the overall mean (change in χ^2^[1] = 412.39, *p *< .001). Furthermore, the model with condition and kinematic properties fit better compared to the model which only included kinematic properties (change in χ^2^[1] = 5.40, *p* = .0202). After accounting for the variance attributed to the different kinematic properties (*p*s < .001), DAF condition had an estimated main effect of about +33 ms (*b* = 32.59 [95% CI: 5.43, 59.76], *t*(1038) = 2.34, *p* = .019). Adding an interaction of kinematic properties and condition did not improve model fit (*p* = .723). Thus, regardless of kinematic property (gesture onset, peak acceleration, ‐velocity, ‐deceleration), DAF beat gestures had positively shifted *D*'s. Thus, the EH can be confirmed for beat gestures with this analysis.

#### 3.5.2 Exploratory analyses EH: Iconic gestures

Similar to beat gestures, there seems to be entrainment to DAF for iconic gestures, as shown by the consistent positive shift of *D*'s (see Table 3 and Fig. 5). A model including kinematic properties fit better than a base model predicting the overall mean (change in χ^2^[1] = 282.51, *p *< .001). Adding condition to the model further improved fit (change in χ^2^[1] = 8.37, *p* = .004). Adding an interaction term for Condition and Kinematic properties did not further improve predictions (*p* = .717). For the final model, after accounting for the variance attributed to different kinematic properties (*p*s < .001), DAF condition had an estimated main effect of about +69 ms (*b* = 68.78 [95% CI: 22.29, 115.26], *t*[1026] = 2.90, *p* = .004). In sum, we find further evidence that iconic gestures produced under DAF are also consistently affected in their timing (similar to beat gestures). Iconic gestures reach their kinematic peaks less rapidly relative to peak pitch (as compared to NO DAF gestures).

### 3.6 Exploratory analyses: Relationship between coupling and entrainment

**Figure 6:**
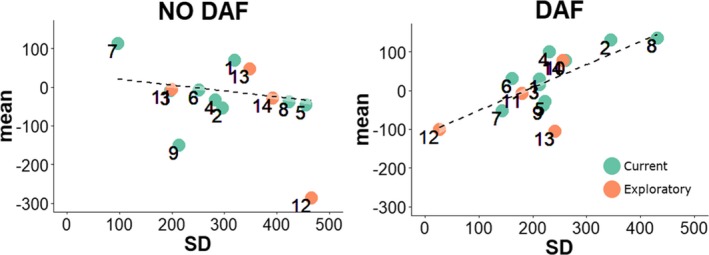
Relationship standard deviation and mean asynchrony (*D* peak velocity). *Note*. Scatter plot of the relationship of the standard deviation with the mean asynchrony for *D* peak velocity with the participants of the current experiment (*N *= 10, numbers 1–10) and the previous exploratory study (*N *= 4, numbers 11–14).

The rationale for the CSH is that gesture–speech synchrony can be modulated so as to resist perturbation by DAF. As such, we would expect that when participants have a higher coupling between gesture and speech (as expressed by lower standard deviations of *D*) there will be a lower entrainment effect (i.e., smaller positive shift for *D*), but only for the DAF condition. We therefore examined the correlations for participants’ standard deviations (*SD*) and mean asynchrony (*M*) of peak velocity *D*, for iconic and beat gestures combined, and for the current and exploratory data combined. As can be seen in Fig. 6, a higher standard deviation for each participants’ *D* peak velocity (as a measure of coupling strength) was associated with a higher positive mean asynchrony of *D* (*r *= .74 [95% CI: .34, 0.91], *t*[12] = 3.78, *p* = .002). This suggests that the higher the coupling strength between gesture and speech (smaller *SD*), the lower the entrainment to DAF (greater positive mean asynchrony). Note, that this inverse relationship holds if we take peak deceleration *SD* and *M*, or only beat gestures or only iconic gestures (or only the current data, excluding the exploratory data). Furthermore, this relation does not hold for the NO DAF condition gestures. Rather, for NO DAF gestures, we find that higher *SD*'s lead to more negative mean asynchronies, (*r* = −.66 [95% CI: −.88, −0.20], *t*(12) = −3.044, *p* = .010); but this effect is possibly carried by two relative outliers as can be seen from Fig. 6. Note that excluding participants from the exploratory study for this analysis does not alter our current interpretation. The *M*–*SD* correlation for DAF excluding the exploratory data remains statistically reliable, *r *= 0.782, *p = *.007.

### 3.7 Robustness analyses

Although we have excellent power, given the densely sampled nature of our data collection, an additional concern might be that our conclusions about the entrainment and the coupling strength hypotheses are carried only by a single participant or by a limited set of participants, which does not reflect the whole dataset. We therefore performed a robustness analyses in which we sequentially excluded all possible combinations of two participants (20% of the total participant number) from the data, and we reran the main analyses. The results of these analyses can be viewed here: https://osf.io/7vxz6/. We obtain for the EH that if we exclude any combination of two participants, that in 100% of the cases (for 45 of the 45^5^ exclusion datasets), the main effect of DAF vs. NO DAF for beat and iconic gestures combined remain significant at *p*'s < .009, after accounting for kinematic properties (same model as used in the EH testing section reported earlier). Thus, there is a positive shift in *D* observed for beat and iconic gestures that are produced under DAF (as compared to NO DAF), regardless of whether we exclude any combination of 20% of the data. We also performed this same type of analyses for the CSH, and we obtained for 93% of the times (42 of 45 exclusion datasets) lower SD's in *D* were reliably found for beat and iconic gestures under the DAF vs. NO DAF condition (*p*'s* *< .011). Note that even for the three exclusion datasets for which we did retrieve non‐significant findings, the *p*‐values were not extremely removed from our original findings, *p*'s < .072. These conservative robustness analyses clearly show that the effects reported here are not carried by a limited set of participants.

### 3.8 Exploratory cross‐wavelet analysis: Gesture (velocity) and speech (ENV)

#### 3.8.1 Introduction to cross‐wavelet analysis

To assess correlated rhythmic activity (i.e., shared periodicities) in gesture and speech on multiple time scales, we performed a cross‐wavelet analysis (Grinsted, Moore, & Jevrejeva, 2004; for good examples see Schmidt, Nie, Franco, & Richardson, 2014; Romero et al., 2018) using the R package “WaveletComp” (Rösch & Schmidbauer, 2014; for a helpful tutorial, see Rösch & Schmidbauer, 2016). Wavelet analysis provides a spectral decomposition of a single time series; that is, it decomposes a complex time series into a set of dominant periodic oscillations continuously across the sampled time period. Wavelet analysis is therefore related to the other spectral decomposition methods (e.g., Fast Fourier Transform). However, a key further specification of cross‐wavelet analysis is that, in addition, it allows for identification of common periodicities^6^ between two time series continuously through time. By decomposing the different periodicities at moments in time that exist *within* time series, such output can be compared *between* time series as well. To this end, cross‐wavelet analysis provides an estimate of degree to which two time series’ periodicities are correlated, and it allows for the estimation of this correlation on different time‐scales that are of interest. The strength of the correlation between the time series across a pre‐defined period range (i.e., time‐scales) is provided by the average coherence (ranging from 0 to 1; no correlation to perfect correlation) and a concomitant *p*‐value for that average coherence. The number of simulations that determine the reliability of the *p*‐value estimates was set at 250 (default = 10).

As mentioned in the introduction, our main aim with this analysis is to assess whether we can detect differences in correlation strength of the periodicities of gesture and speech on multiple nested timescales. Given that we are dealing with spoken language and physical movement, the following theoretically relevant timescales can be proposed. We set the period range at which average coherence was computed as to include possible periodicities at the timescale of a clause and a sentence (~2–6 s; i.e., 0.5–0.16 Hz or a period of 6 s), the gesture (~0.5–2 s; 2–0.5 Hz or a period of 1 s), up to faster frequencies to accommodate for the average length of a syllable (~200–500 ms; i.e., 5–8 Hz or a period of 0.2 s).

**Figure 7:**
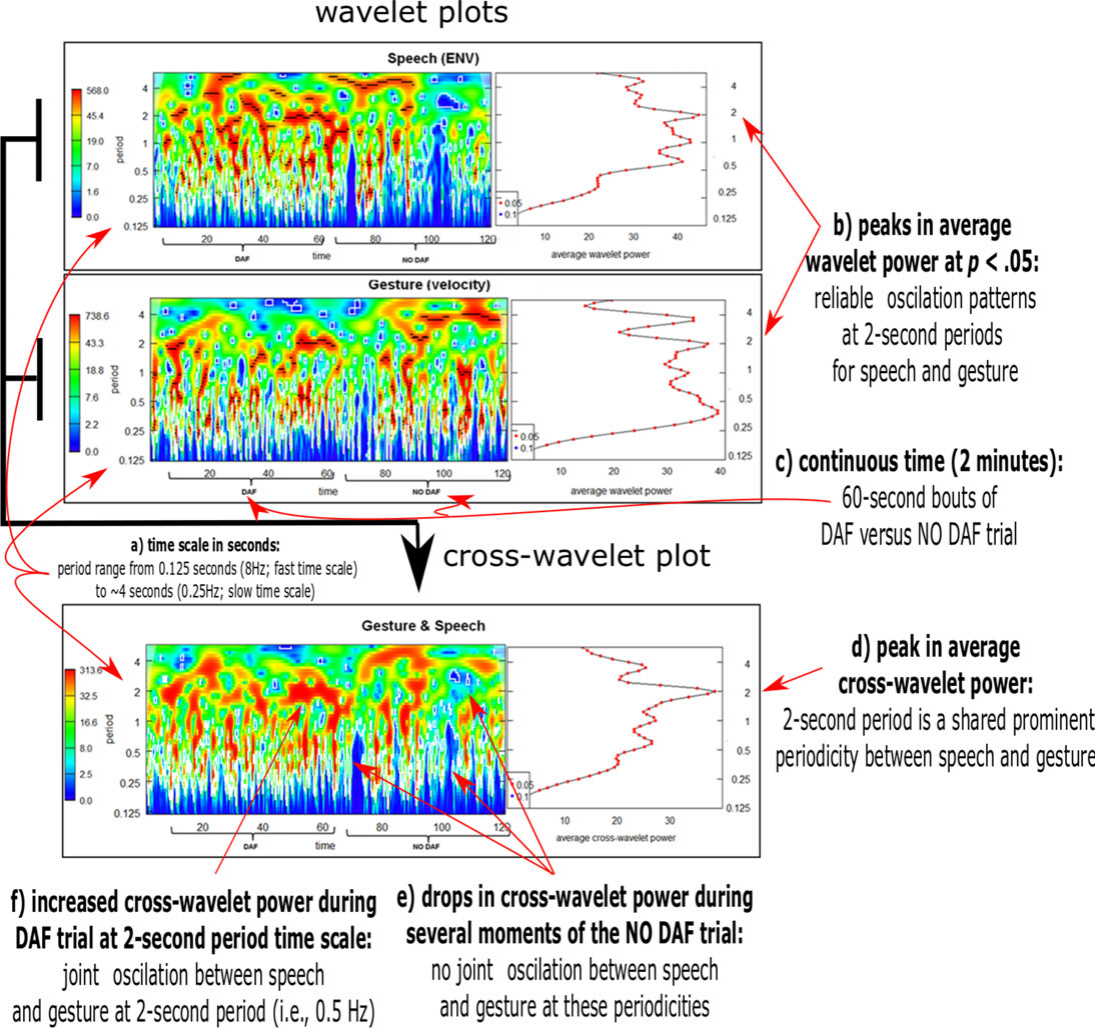
Wavelet and cross‐wavelet analyses plots for participant 1. *Notes*. The top and middle panels show wavelet power plots. Time is on the horizontal axis (see note ***c***). The vertical axis shows the different time scales expressed as “period” (Note to convert to Hz = 1/period; i.e., period 0.5 = 2 Hz, period 4 = 0.25 Hz.). Vertical axes show the time scales from 0.125 s to ~4 s periods (see note ***a***). Darker red areas indicate higher power indicating stronger periodicities at that time scale for that time segment. The right panels show the average power (horizontal axis) for each time scale (vertical axis) (see note ***b***). The red dots indicate which periodicity is statistically reliable at *p* < .05. The lower panel shows the cross‐wavelet results. Darker red areas indicate higher cross‐wavelet power for shared periodicities at that time scale for that time segment (see note ***e*** and note ***f***). The bottom right panel shows a summary of the average cross‐wavelet power for each timescale (see note ***d***). The horizontal axis represents the average cross‐wavelet power indicating a degree of shared variance explained for a particular periodicity. Notice for example, that for both speech and gesture there is a peak at 2‐s periods (see note ***b***), which produces a shared peak at 2 s in cross‐wavelet power (see note ***d***). This indicates that gesture and speech seem to oscillate with a joint period of 2 s or a frequency of 0.5 Hz (for this participant). Note, however, that the shared periodicities are very complex and show varied coupling across time and period (except periods <0.20 s, i.e., <200 ms).

To provide an example of how cross‐wavelet analysis decomposes and relates two time series, Fig. 7 shows a visual presentation of a cross‐wavelet analysis for the time series of participant 1 for two consecutive “trials” of 60 s, starting with the first DAF trial (after 30 s warm up) and following the NO DAF trial of equal length. The cross‐wavelet analysis plot provides periodicities for the velocity and ENV time series separately (represented by the first two upper panels). For these “univariate” wavelet analyses, an estimate of the spectral “power” is provided through time as well as an average power over time (right panels). Power expresses the amount of variance that is explained by a particular pure periodic signal (e.g., 2‐s period) in the complex time series. Thus, power allows you to compare which periodicities are more dominant in the complex time series. Similarly, in the bottom panel the “cross‐wavelet” analysis is shown which provides the strength of the *shared* periodicities as quantified by the cross‐wavelet power. Cross‐wavelet power expresses the relative strength of periodicities that are shared between two time series (represented by the bottom panel with more red‐colored areas denoting higher cross‐wavelet power for shared periodicities between gesture and speech).

To interpret the *cross‐wavelet* plot results, we can further assess the bottom panel of Fig. 7: We see that for this participant there seems to be a slight drop in the correlations of the speech (ENV) and gesture (velocity) time series (see note ***e*** in Fig. 7), as expressed by the lower cross‐wavelet power levels for the NO DAF trial segment (as compared to the DAF segment); visually represented as a decrease in red‐colored areas (and an increase in blue‐shaded areas). If generalizable, this supports the idea that gesture and speech are more tightly coupled under DAF. For this participant, a reliable common frequency of speech and gesture is observed for the 2‐s period, which indicates that movement and speech are coupled over parts of a sentence. If gesture–speech coupling strength is affected by DAF, we thus might find such effects on several time scales. However, to formally test this, we reconstructed for each participant a time series for speech and gesture produced under DAF vs. NO DAF by concatenating alternating trials into a single continuous DAF versus NO DAF time series per participant. The reconstructed time series of gesture and speech was standardized for each trial (excluding possible artifacts of difference in amplitude levels between DAF vs. NO DAF trials, while preserving temporal structure within trials^7^ ). We subsequently entered these time series into a cross‐wavelet analysis from which we obtained the “average coherence” and “*p*‐values for the average coherence” per condition and per participant across the predefined timescales (0.2–6 s periods). Average coherence provides a summary statistic of the correlation strength of the time series over the pre‐defined time scales (i.e., different periods), its output ranging from 0 (no correlation) to 1 (perfect correlation). The conventional *p*‐values provide a reliability estimate by testing against the null hypothesis that there is no joint periodicity.

The final cross‐wavelet analyses were performed to test whether coupling strength differences between gesture and speech were present in the form of correlated periodicities (average coherence) between amplitude envelope of speech (ENV) and the velocity of the hand movements. Importantly, in addition to assessing the effect of condition on average coherence, we wanted to assess whether differences in coherence for condition were pronounced at particular time scales. We therefore constructed a three‐level categorical variable to capture three theoretically relevant time scales (see for a similar procedure Schmidt et al., 2014). First, on the basis that an average syllable length is about 200–300 ms (Turvey, 1990) we will define a “fast” syllable‐level time scale (0.2–0.5 s periods). Given that we obtain in the current dataset that gestures take about 700 ms to complete (and some may last as long as 2 s), we will define a “medium” gesture‐level timescale (0.5–2 s; 1 Hz or a period of 1 s). Finally, although the average sentence length is difficult to judge (given its inherent variability), we estimate most sentences to not take longer than 6 s and we will use this as the upper bound for the slowest timescale of interest, that is, “slow” sentence‐level timescales (which included coherence levels for 2–6 s periods). Note that our definitions of the timescales are given to provide a sense of the dominant timescale for each phenomenon, but clearly there is a distribution of times for each modality around these center values.

**Figure 8:**
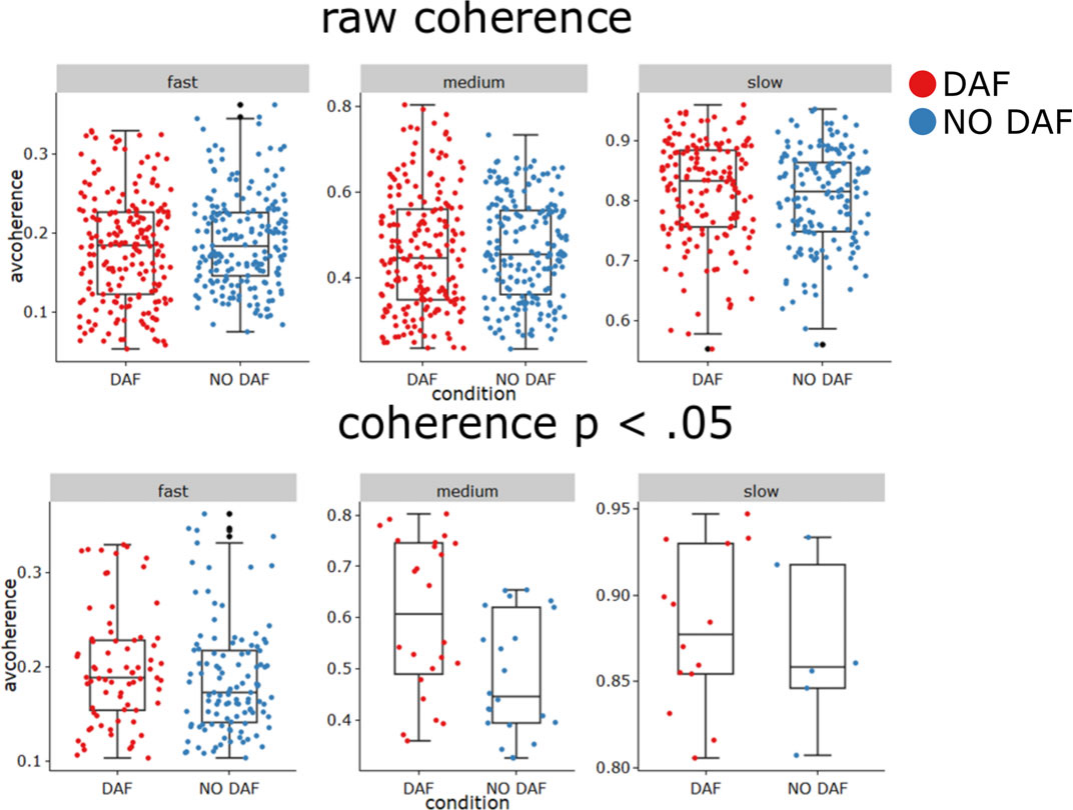
Average coherence levels for fast to slow time scales under DAF vs. NO DAF. *Notes*. Box‐ and jitter‐plot for the average coherence levels per time scale. The colored “jittered” dots provide individual coherence data points. The upper panel shows the raw coherence levels, and the lower panel shows only the coherence levels that were statistically reliable at *p* < .05. Note that reliable periodicities are observed on all time scales. “Fast” time scale indicates average coherence between ENV and velocity for periods relevant to syllable completion times (200 ms to 500 ms); “Medium” time scales are most relevant to gestures (500 ms to 2 s); “Slow” time scales are most relevant to clauses and sentences (2–6 s).

Having identified timescales of interest, we performed two separate analyses. First, we assessed raw average coherences per participant and per condition for all periodicities. Second, we performed an analysis where we compared coherence strengths that were found statistically reliable (*p* < .05). Fig. 8 provides a visual overview of the average coherence levels for each time scale.

To test whether differences in raw average coherence levels (Fig. 7 upper panel) exist for DAF vs. NO DAF trials, and whether such differences are more pronounced on specific time scales, we performed a mixed regression (with a random intercept for each participant) with average coherence as the dependent variable. As predictors, we included time scale (model 1), timescale and condition (model 2), as well as finally adding an interaction of condition and timescale (model 3). However, in all the models, condition was not a reliable predictor of raw average coherence (*p*'s > .71). Only model 1 with time scale as predictor for average coherence showed significantly improved fit relative to a model predicting the overall mean (change in χ^2^[1] = 2,291.31, *p *< .001). Slower time scales had higher raw coherence levels than fast time scales (fast < medium < slow; all comparisons *p*'s* *< .001).

However, the raw coherences might contain correlation estimates that are not reliable. Indeed, it is common for these types of continuous bivariate time series analyses to treat coherences as meaningful indicators of reliable relations given some arbitrary cutoff (e.g., see Danner et al., 2018). Instead of deciding on a cut‐off based on the correlation coefficients, the current cross‐wavelet analyses conveniently provide reliability estimates (*p*‐values) for coherence estimates by testing against the null hypothesis that there is no correlated periodicity. As such, we can filter our coherence estimates, by considering only coherence estimates that were found reliable at *p* < .05, and compare possible differences across conditions for those statistically reliable periodicities (Fig. 6 lower panel). Note that this drastically reduces the amount of data points as only 22% of the coherence estimates reached the *p* < .05 threshold. A model containing time scale as a predictor for statistically reliable average coherence had improved fit, relative to a model predicting the overall mean (change in χ^2^[1] = 525.227, *p* = .001). Adding condition as predictor to the model improved the fit (change in χ^2^[1] = 11.938, *p *< .001). Consistent with the overall findings, the average coherence across the time scales was lower for the NO DAF trials as compared to the DAF trials (*b *= −0.036 [95% CI: −0.056, −0.036], *t*(187) = −3.510, *p* < .001). Adding an interaction of time scale and condition further improved the model compared to the previous model containing only condition and period (change in χ^2^[1] = 22.78, *p *< .001). The final model revealed that the effect of increased coherence levels for DAF was carried only for the medium time scale (0.5–2 s), which in the model containing interactions is reflected by statistically reliable interaction of condition × medium time scale (as compared to the average coherence for the fast time scale), *b *= −0.124 [95% CI: −0.174, −0.074], *t*(182) = −4.822, *p *< .001), but no reliable interaction of condition x slow time scale, *b *= −0.009 [95% CI: −0.085, 0.007], *t*(182) = −0.239, *p *= 0.811). In sum, the current continuous time series analyses support our earlier parametric findings. We show that for statistically reliable periodicities there is higher coherence between gesture and speech for the DAF vs. NO DAF condition, especially for periodicities around 0.5–2 s. It is further important that statistically reliable periodicities were obtained at slower (2–6 s) and fast time scales as well (200–500 ms), suggesting that gesture and speech are coupled on multiple nested time scales.

## 4 Discussion

**Figure 9:**
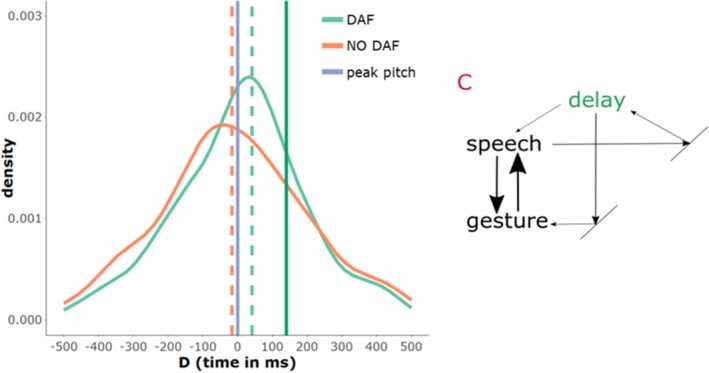
The dynamically coupled model and the summary data. *Notes*. The dynamically coupled model as shown in Fig. 1 is shown here on the right. On the left is a summary of the actual data obtained for peak‐velocity and peak pitch (a) synchronies (*D*) for all beat and iconic gesture events that occurred during this study. It can be seen that the distribution of *D* for gestures produced under DAF is more peaked, indicating that gesture and speech are more synchronized under DAF (supporting the coupling strength hypothesis). Secondly, gestures produced under DAF consistently have a slight offset, such that gestures are slightly delayed wherein peak velocity was more closely aligned with the delay of speech (supporting the entrainment hypothesis).

In this study, we assess how strongly gesture and speech are coupled by perturbing the speech production system. We perturbed the speech production system by providing participants with a delayed auditory feedback of their speech of 150 milliseconds, which leads to speech disfluency. We made particular model‐based predictions that variably state how gesture and speech are related (see Fig. 1). First, we introduced the ballistic model which states that gesture and speech are decoupled at late stages of execution. This ballistic model predicts that speech will lag behind on gesture when speech production is impaired, as there is no means of readjusting the gesture stroke to accommodate speech (leading to considerable gesture–speech asynchronies). Second, we introduced the sparsely coupled model, which states that there is bidirectional coupling between gesture and speech which allows continuous recalibration of gesture and speech. The sparsely coupled model predicted that gesture‐speech synchrony would be maintained when speech was perturbed, as gesture can readjust and slow down if speech disfluencies occur. Finally, the currently favored dynamically coupled model (also see Fig. 9) suggests that gesture‐speech coupling strength is something that is intensified when speech disfluencies occur as it allows for gaining stability under interfering conditions. This model further predicts that the gesture system is (next to speech) sensitive to the auditory delay of speech. Namely, gesture activity will inadvertently entrain to the auditory delay, leading to gesture‐speech asynchronies that indicate gesture's synchronization with the delay of speech.

Our results indicating that gesture‐speech synchrony is present under DAF leads us to conclude that gesture and speech are indeed bi‐directionally coupled (discounting the ballistic model). This supports McNeill's (1992) pioneering descriptive work, as well as more kinematically detailed research on non‐spontaneous gesturing (Chu & Hagoort, 2014; Rusiewicz et al., 2014).

This study, however, extends this previous research by also showing that gesture and speech are *more tightly* coupled under DAF. Namely, our exploratory analyses suggest that all gestures (i.e., beat and iconic) produced under DAF (vs. NO DAF) were more tightly coupled to peak pitch as indicated by smaller standard deviations for *D*'s for all key kinematic anchor points. In other words, gestures were less variably aligned with peak pitch under DAF. Initially we predicted that increased gesture‐speech coupling would also be translated into more absolute differences in asynchronies, as this was also originally found in an exploratory study with beat gestures (Pouw & Dixon, unpublished data). However, we could not replicate this confirmatory prediction in the current analysis. We think, however, that the exploratory analysis provides strong additional evidence for the coupling hypothesis as it more directly addresses the question whether distributions of *D* are more or less peaked (as a measure of coupling strength), as opposed to looking at absolute deviances from peak pitch. Furthermore, looking at absolute deviances of peak velocity from peak pitch as a proxy for gesture‐speech coupling strength, is in hindsight not the ideal test because there is also an entrainment effect found which inevitably leads to more absolute deviances in the DAF condition (as can be seen in Fig. 9). Thus, given that the more adequate exploratory analyses show clear stable effects, we conclude that across the board the CSH is supported; gesture–speech coupling is strengthened when the speech system is perturbed by an interfering signal (i.e., DAF).

The exploratory cross‐wavelet analysis further corroborate the CSH by showing that the shared temporal structures of gesture and speech had a lower correlation (average coherence) for statistically reliable shared periodicities, which was especially pronounced for time scales at the level of a gesture completion (0.5–2 s). As such, this analysis supports our main findings by indicating that increased gesture‐speech coupling is detectable in shared periodicities as well. We also provide evidence that shared periodicities are statistically reliable at slower timescales (2–6 s), providing novel quantitative evidence for the theoretical claim (Kendon, 2004; McNeill, 2005) that gesture and speech are coordinating on multiple time scales (e.g., a gesture‐speech event can be nested in a sequence of gesture‐speech events), although we do not find evidence that DAF is disrupting gesture‐speech coupling on these slower timescales.

Secondly, we find strong evidence that despite gesture‐speech system's resistance to perturbation, gestures seem to entrain to the DAF signal. Both beat gestures’ and iconic gestures’ kinematic anchor points were positively shifted. It is as though gestures’ kinematics is attracted toward the delayed auditory event which occurs after the actually produced peak pitch. Furthermore, we find a promising indication that higher coupling strength (lower standard deviations for *D*'s) is associated with lower entrainment effects, suggesting that increased coupling strength may reduce interference of DAF on gesture.

To note, we are confident in the presence of the current DAF effects in the *current sample*, as we yielded such findings from multiple analyses techniques, additional robustness analyses, and a pre‐registered replication of earlier exploratory results. However, given that we base our conclusions on only a small number of participants (*N* = 10 and *N* = 4 for the confirmatory and exploratory study, respectively), we should be cautious in generalizing the currently obtained dynamics in the wider population. More research and methodological innovations in multimodal research are needed that can take up such claims of generalizability.

### 4.1 Theoretical implications

We propose based on the dynamically coupled model that DAF *of speech* affects both speech and gesture production. At first blush this is counterintuitive, because the DAF of speech only constitutes inherently relevant information to the speech production system. However, that DAF of speech affects gesture is less surprising if we consider the evidence that gesture is strongly coupled to the speech system (McNeill, 2005); thus, effects of speech production *are* inherently meaningful for the gesture system. Furthermore, there is a possibility that the entrainment effect is also not dependent on the inherent meaningfulness of the DAF signal per se. The differences in how DAF affects gesture and speech may rather be explained by the differing intrinsic or “preferred” frequencies of gesture and speech which, therefore, lead to different entrainment relations to the DAF signal. This is a well‐known phenomenon for coupled oscillator systems called *detuning* (see p. 11; Pikovsky, Rosenblum, & Kurths, 2001). Considering that the average duration for a syllable is about 200 ms, the current DAF of 150 ms presents an interfering out‐of‐phase signal. Yet a gesture event completes its movement at about 700 ms and is likely, therefore, to entrain differently than speech to the DAF of speech. Indeed, it has been shown that the rate at which rhythmic tapping is performed will affect the degree to which those actions are sensitive to a DAF manipulation (Finney & Warren, 2002); tapping at an interval of 250 ms (vs. 400 ms) will maximally be interrupted by a roughly similar‐valued DAF of the tapping action (250 ms rather than 400 ms delay). Therefore, the entrainment effect could equally be accommodated by theories that aim to explain effects of DAF for rhythmic sensorimotor behavior *in general* (rather than DAF affecting speech in a special way), such that a DAF signal oscillates at a frequency that can *impair* (speech) or *attract* (gesture) periodicities in the behavior which the DAF shadows (Finney & Warren, 2002; Howell, Powell, & Khan, 1983).

That gestures are more tightly (less variably) coupled to speech under DAF may be an important lead for understanding the cognitive function of beat and iconic gesture–speech synchrony that goes beyond its obvious role in communication. This modulation of gesture–speech synchrony is also particularly interesting, because there is a host of research showing that DAF leads to *less* synchrony in rhythmic tapping tasks, where one needs to tap with an external rhythm or continue tapping after the external signal has stopped (e.g., Finney & Warren, 2002; Repp, 2005). We think that the key difference is that gesture with its own intrinsic dynamics (and thus different entrainment to DAF) can be utilized to resist DAF in speech production. We speculate that under conditions where speech is made difficult due to DAF, the increased coupling of gesture and speech may allow the gesturer to maintain her own preferred “oscillation rate” (i.e., maintain rhythmic speech). In keeping with the coupled oscillator parlance, increasing the coupling strength between two oscillators diminishes the relative coupling strength with an interfering third oscillator. That active intentional mechanisms play some role in gesture‐speech synchrony resonates with findings from a finger‐tapping paradigm. Treffner and Peter (2002) found that tapping‐speech coupling can be affected by explicitly focusing attention on speech or finger tapping (Treffner & Peter, 2002). Furthermore, effects of attention were especially pronounced when the tapping‐speech coordination was in a less stable regime (i.e., tapping out of phase with speech), which suggests that attentional mechanisms come into play when, and may help to persist in, performing a coordination that is inherently more unstable (e.g., gesture–speech under DAF). Although the precise effects on gesture‐speech synchrony on speech go beyond the current paper and should be further studied in the present context, our findings suggest that stability through synergy may be a key mechanism for understanding the cognitive function of gesture. This resonates with dynamical systems perspectives on gesture‐speech coordination (Iverson & Thelen, 1999; Jonge‐Hoekstra, Van der Steen, Van Geert, & Cox, 2016; Rusiewicz, 2011; Rusiewicz & Esteve‐Gibert, 2018), as well as with David McNeill's (1992, 2005) lifework on the Growth‐point theory of gesture.

In sum, the current findings suggest that gesture‐speech synchrony is modulated so as to maintain stability under perturbation. It can therefore be related to research indicating that gesture may help listeners understand a *speaker*'s message when auditory information is degraded (e.g., trying to have a conversation at a crowded cocktail party; Drijvers & Ozyurek, 2018), and speaking more rhythmically may help listeners’ understanding when the speech signal is more noisy (Wang, Kong, Zhang, Wu, & Li, 2018). Conversely, gesture‐speech synchrony may help the *gesturer* to keep a preferred prosodic rhythm that is more difficult to maintain without bodily synchronization, especially when speech is perturbed due to an interfering signal (e.g., loud music during a party). We suggest that stability by means of sensorimotor synchronization may be an important expansion of the intra‐cognitive functionality of gesticulation that has so far been largely overlooked (e.g., De Ruiter, 2000; Goldin‐Meadow & Beilock, 2010; Hostetter & Boncoddo, 2017; Kita, Alibali, & Chu, 2017; Pouw, De Nooijer, Van Gog, Zwaan, & Paas, 2014; ).

## Open data and pre‐registration

This study is pre‐registered at the Open Science Framework. The pre‐registration, experiment code, (raw) anonymized quantitative data, and analyses scripts supporting this study are available at https://osf.io/pcde3/.

## Funding

This research has been funded by The Netherlands Organisation of Scientific Research (NWO; Rubicon grant “Acting on Enacted Kinematics,” grant no. 446‐16‐012; PI Wim Pouw).

## Notes

1 For statistical power analyses: https://osf.io/5h3bx/.

2 Note that for present purposes, *metaphorical* gestures (McNeill, 2005) will not be distinguished from, and fall under the category of, the iconic gesture label in this study.

3 We found that 33 Hz resulted more smooth derivative time series (i.e., velocity) as compared to the 10 Hz cut‐off as initially proposed in the pre‐registration.

4 For all mixed regression models reported throughout the paper, we used maximum likelihood estimation.

5 There are 45 unique combinations of two participants that can be excluded.

6 There are other functionalities such as assessing leading/follower relations between periods (i.e., “relative phase relations”). However, in the current paper we will restrict ourselves to the gross coupling between gesture and speech.

7 We also reran another set of analyses where we computed coherence statistics for each trial separately instead of concatenating the DAF and NO DAF time series. Our current interpretations are not affected by these different approaches; for an overview of this analyses, see https://osf.io/pdbk2/.
